# 5-Aminosalicylic Acid Loaded Chitosan-Carrageenan Hydrogel Beads with Potential Application for the Treatment of Inflammatory Bowel Disease

**DOI:** 10.3390/polym13152463

**Published:** 2021-07-27

**Authors:** Cristina Elena Stavarache, Adi Ghebaur, Sorina Dinescu, Iuliana Samoilă, Eugeniu Vasile, George Mihail Vlasceanu, Horia Iovu, Sorina Alexandra Gârea

**Affiliations:** 1 Advanced Polymer Materials Group, University Politehnica of Bucharest, 1-7 Gh. Polizu Street, 011061 Bucharest, Romania; cristina.stavarache@upb.ro (C.E.S.); adi.ghebaur@upb.ro (A.G.); vlasceanu.georgemihail@yahoo.ro (G.M.V.); horia.iovu@upb.ro (H.I.); 2 C. D. Nenitescu, Centre of Organic Chemistry, 202-B Spl. Independentei, 060023 Bucharest, Romania; 3 Department of Biochemistry and Molecular Biology, University of Bucharest, 050095 Bucharest, Romania; sorina.dinescu@bio.unibuc.ro (S.D.); iuliana.samoila@bio.unibuc.ro (I.S.); 4 Department of Science and Engineering of Oxide Materials and Nanomaterials, Faculty of Applied Chemistry and Material Science, University Politehnica of Bucharest, 1-7 Polizu, 011061 Bucharest, Romania; eugeniuvasile@yahoo.com; 5 Faculty of Medical Engineering, University Politehnica of Bucharest, Gh Polizu 1-7, 011061 Bucharest, Romania; 6 Academy of Romanian Scientists, 54 Splaiul Independentei, 050094 Bucharest, Romania

**Keywords:** chitosan, k-carrageenan, 5-aminosalicylic acid, ionic gelation technique, drug delivery system

## Abstract

The aim of our work is to prepare mucoadhesive particles with biopolymers and 5-Aminosalicylic acid (5ASA) using the ionotropic gelation technique to ensure a controlled drug release at the colon level with potential applications in the treatment of intestinal bowel disease (IBD). The preparation of particles through the crosslinking of Chitosan (CS) with sodium tripolyphosphate (TPP) using different mass ratios and the influence of the k-Carrageenan (kCG) layer were studied. UV–VIS spectrometry was employed to assess encapsulation efficiency and drug release profile of 5ASA. The particles were investigated using FT-IR spectrometry for chemical characterization and the DLS results highlighted a monodisperse particle size distribution. The morphology of the polymeric beads was investigated using micro-computer tomography (µCT) and Scanning Electron Microscopy (SEM). Particles based on Chitosan and k-Carrageenan were able to incorporate and preserve 5ASA in an acidic and alkaline medium. The 5ASA loaded polymeric particles obtained after immersion for 1 h in kCG solution exhibited the lowest release rate in pH = 1.2. Biocompatibility studies performed on all of the particles displayed a good viability for the CCD 841 CoN cells and low cytotoxicity. All of the results have shown that these new biomaterials could be a versatile platform of targeted carriers with potential applications in inflammatory bowel disease treatment.

## 1. Introduction

Inflammatory bowel disease (IBD) is a chronic and nonspecific inflammatory disorder mainly affecting the large and small bowel. The two major types of IBD are Crohn’s disease (CD) and ulcerative colitis (UC). 5-Aminosalicylic acid (5ASA), alongside with methotrexate, corticosteroids, and sulfasalazine, is one of the current drugs used in a conventional treatment plan for IBD as a result of its anti-inflammatory and antioxidant effects on the swelled intestine [1,2,3]. Even though it is commonly used in IBD treatment, high doses of 5ASA are necessary, which leads in time to side effects because it is rapidly and extensively absorbed by the mucosa of the upper gastrointestinal tract, which hinder the therapeutic action in the proximal colon. Therefore, it is essential to have an efficient absorption and to maximize the release of the drug in colonic mucosa to minimize the toxic effect of the anti-inflammatory therapy [4,5]. In order to protect the drug from the acidic environment of the stomach and to achieve colon specific drug release, different approaches have been employed, such as time dependent systems, enzyme-dependent and pH-dependent coatings as well as biodegradable polymers matrices, hydrogels, and pro-drugs [2,6,7].

A targeted drug delivery to a distinct location in the body has the advantage of prolonging and controlling the drug release to avoid overdose. For a drug delivery system that is able to target the colon, the pharmaceutical compound has to cross the abdominal region and the small intestine to the required destination in a manner that keeps it more or less intact [8,9]. Biopolymers, such as polysaccharides, have been used as possible encapsulation materials due to their biocompatibility, non-toxicity, mucoadhesive properties, plentifulness in nature, and low price. Natural hydrophilic ionic biopolymers such as alginate, pectin, chitosan, and k-carrageenan are widely used in pharmaceuticals, in the food industry, and in medicine because of their biocompatibility and biodegradability properties [10,11,12].

Chitosan (CS), a cationic, hydrophilic polysaccharide extracted from chitin, is composed of N-acetylglucosamine units that are β-(1–4) linked with D-glucosamine repeating units. Due to its cationic substrate, the ^+^NH_3_ groups, CS may interact or form a complex bond with other anionic surface. The biocompatibility, biodegradability, low-toxicity, and mucoadhesiveness of this biopolymer are responsible for its recommendation for use in medicine and in the pharmaceutical industry in drug release applications and tissue engineering. Moreover, CS promotes cellular adhesion, and it allows oxygen permeability, which makes CS a perfect biomaterial for artificial tissue preparation and tissue regeneration [13,14,15]. CS particles that are crosslinked with sodium tripolyphosphate (TPP), a non-toxic polyanion, present important results in delivering papain, heparin, and the absorption of metals ions [13,16]. Additionally, ciprofloxacin loaded alginate beads and coated with chitosan layer were prepared using the gelation method with TPP. After coating the alginate surface with the muchoadesive biopolymer, CS reduces the drug release in the acidic medium of the stomach and at a higher pH value (6.5), and the ciprofloxacin had a low release [17].

Chitosan has been successfully employed to obtain 5ASA nanoparticles via the ion gelation method using TPP as a crosslinking agent [18]. To improve the loading efficiency of 5-ASA, which is a hydrophobic drug, Tang and et al. [19] incorporated hydroxypropyl-β-cyclodextrin into CS nanoparticles obtained using TPP solution to improve the loading efficiency.

Carrageenan (CG) is another marine-derived anionic, hydrophilic biopolymer extracted from marine algae *Rhodophyceae*. Carrageenan presents the general name for a class of high molecular weight sulphated polysaccharides. They consist of alternative galactose and anhydrogalactose units linked by a glycosidic junction. Carrageenen is classified in three important forms (kappa, iota, and lambda) determined by the position and number of its ester sulfate groups. Of these three types of CG, kappa (k) and iota (τ) exhibit gel formation ability, and they have the very interesting capability of forming gels after cooling, even at room temperature, as a result of developing a three-dimensional structure made of the crosslinking of the neighboring SO_4_^2−^ groups from the double coil-helix of polymeric chains. CG can be converted into gels with the help of ions (Li^+^, K^+^, Na^+^, Ca^2+^). Gels prepared using τ-CG are more elastic and softer, while those obtained using k-CG are more firm, and because of that property, k-CG can be used in controlled released technology [14,20,21,22,23]. Because of its very unique and interesting physicochemical properties, CG can prolong the release of pharmaceutical substances and increase drug bioavailability [21] and provide suitable cell proliferation and adhesion [24,25]. CG is successfully used in the food industry as a food additive due to its thickening properties [24], and it shows anticoagulant, antiviral, and antitumoral activities [26].

However, the high solubility of CS in the stomach limits its application as a host designed for drug release protection during their pass to the small intestine, and therefore Sun et al. [27] developed a three-component bead made from alginate, CS, and kCG via electrostatic interaction/ionic gelation technique.

Electrostatic interaction between groups with opposite charges of the two biopolymers generate a polyelectrolyte complex bicomponent that can manufacture hydrogel beads as a controlled drug delivery system [14,27]. CG–CS polyelectrolyte complexes can be obtained in various forms such as films, microcapsules, microspheres, and gels to be used as drug delivery agents and in cosmeceuticals and nano-layered coatings [21].

Using the emulsification method, CS/λ-CG double coated 5ASA loaded alginate particles were obtained to improve particle strength in both the gastric and intestinal environments and a better controlled drug release [28]. The CS-GC polyelectrolyte complex has been employed for the improved drug delivery of sodium diclofenac [29], theophylline [30], and diltiazem hydrochloride [31].

There have been studies that have assessed the anti-inflammatory activity of the CS/kCG complex in inducing colitis in mice at different concentrations, showing their protective role in gastrointestinal tract [21,26].

The aim of our study is to formulate a colon targeted bi-component bead using 5ASA by coating the CS-TPP particles with a kCG layer employing electrostatic interaction between the two biopolymers. Microparticles synthesized from the ionic crosslinking of CS with non-toxic TPP show an improved drug loading efficiency, which is based on previous studies [13]. The objectives were to evaluate the effects of the CS–TPP ratio and crosslinking time on the particle size, morphology, encapsulation efficiency and drug release profile. The purpose is to improve the drug release at the colon level with potential applications for the treatment of inflammatory bowel disease (IBD).

## 2. Materials and Methods

### 2.1. Materials

Medium-molecular-weight chitosan, (CS), k-Carrageenan (kCG), and 5-Aminosalicylic acid (≥99%) were purchased from Sigma-Aldrich, St. Louis, MO, USA. Calcium chloride anhydrous powder (CaCl_2_) was purchased from Merck, Darmstadt, Germany. Sodium tripolyphosphate (TPP), sodium chloride (NaCl), potassium chloride (KCl), hydrochloric acid ≥37% (HCl), and potassium phosphate monobasic (KH_2_PO_4_) were supplied by Sigma-Aldrich, St. Louis, MO, USA and sodium hydroxide pellets (NaOH) were supplied by Riedel-de Haën, Seelze, Germany.

### 2.2. Hydrogel Beads Preparation

**Figure 1:**
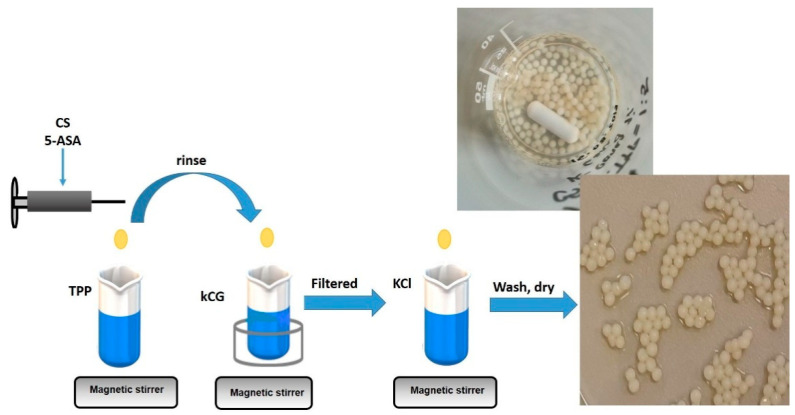
Synthesis route of CS-kCG drug loaded hydrogel beads.

The preparation of 5-ASA loaded CS-TPP-kCG hydrogel beads involved two steps (Figure 1). The ionic gelation technique employs an ionic interaction between the positively charged amino groups of CS and the sodium tripolyphosphate (TPP) anions, which acts as chitosan crosslinker, followed by polyelectrolyte complexation with the help of an electrostatic interaction between the oppositely charged groups from the two biopolymers. Additionally, in the last step of the synthesis, the particles were immersed in KCl solution. The preparation method of the beads was a modified method from the work and studies of S. Rodrigues et al. [14] and Qing-Xi Wu et al. [15]. S. Rodrigues et al., prepared nanoparticles based on the electrostatic interaction of the CS with kCG followed by ionic gelation of CS with TPP, without studying the effect of any drug incorporation, only the ratio between the nanoparticle components. The microparticles designed for the 5ASA encapsulation by Qing-Xi Wu et al. [15] were manufactured from CS and sodium cellulose sulfate with sodium polyphosphate as a crosslinking agent to produce a drug release system triggered by enzymes. The chemical 5-fluorouracil was incorporated by Sun et al. [27] in a drug delivery system made from alginate, chitosan, and k-carrageenan by coating the microbeads prepared from crosslinking alginate with CaCl_2_ with dual layer of CS and kCG. Potassium chloride was used to complete the crosslinking process of the second biopolymer layer.

Briefly, 5 mg of 5ASA were dissolved in 5 mL of 2% CS solution (prepared in 1.1% acetic acid) overnight using magnetic stirring to obtain a drug–chitosan solution. The mixture was extruded drop wise through a syringe with various volumes of 5% TPP to gain theoretical CS–TPP weight ratios of 1/5 and 1/10 (*w*/*w*). The hydrogel beads were kept for 15, 30, and 60 min in the TPP solution to become hard. After the end of the crosslinking period, the beads were settled, filtered, and covered with kCG by immersing them into a 1% kCG solution for 1, 3, and 6 h in a heated water bath at 38–39 °C. The final step of the synthesis route was to put the CS-TPP-kCG drug load beads into a 0.3 M KCl solution for 1 h to complete the process due to the intermolecular glue-like effect of the K^+^ ions [23,32]. The hydrogel beads were then washed with distillated water and air-dried for 4 days. The same protocol was followed for the blank CS-TPP-kCG hydrogel beads. The 1% kCG solution was prepared by dissolving the polymer at 65 °C.

### 2.3. Hydrogel Beads Characterization

#### 2.3.1. Fourier Transform Infrared (FT-IR) Spectrometry

The structural study of the hydrogel beads and the drug was performed on a FTIR-ATR Bruker VERTEX 70 spectrometer (Bruker, Billerica, MA, USA). The spectra of the dried samples were recorded between in the 4000–400 cm^−1^ wavenumbers range, with 4 cm^−1^ resolution and 32 scans for each spectrum at room temperature.

#### 2.3.2. Biocompatibility of the Hydrogel Beads

Hydrogel beads were tested for their biocompatibility in contact with intestinal epithelial cells. Following a sterilization procedure using exposure to UV light, the materials were put in contact with intestinal epithelial cells from the CCD 841 CoN cell line (ATCC CRL-1790, ATCC, Manassas, VA, USA). Cells were previously seeded at a density of 1.5 × 104 cells/cm^2^ and allowed to adhere for 24 h prior to exposure to materials. Contact was maintained between the cell culture and beads in standard culture conditions (37 °C, 5% CO_2_ and humidity) for 7 days, during which time cell viability (MTT assay), material cytotoxicity (LDH assay), and fluorescent microscopy staining (Live/Dead assays) were assessed at 2 and 7 days.

The viability MTT assay was conducted by incubating the cultures for 4 h in the MTT solution of 1 mg/mL concentration. The resulting formazan crystals were solubilized in isopropanol, and the violet solution was measured at 550 nm using spectrophotometry. The cytotoxicity LDH assay was performed using the TOX7 kit (Sigma-Aldrich Co, Steinheim, Germany), following manufacturer’s instructions. A 1:1:1 ratio between the 3 kit components was used, which was mixed with the culture media collected after 2 and 7 days of culture from the samples. The solution was incubated for 20 min in the dark and then was stopped with 1 N HCl. Using spectrophotometric measurements, the resulting pink solution was quantified at 490 nm. The qualitative Live/Dead assay was performed using a Live/Dead kit (Invitrogen, Life Technologies, Foster City, CA, USA), which contained two fluorescent components—calcein AM, which stained the live cells in green, and ethidium bromide, which stained the dead cells in red, respectively. After being exposed to the Live/Dead solution for 30 min, the culture was examined using confocal microscopy (Zeiss LSM 710, Jena, Germany), and the images were processed using Zeiss Zen software (2010 Software Version, Carl Zeiss AG, Jena, Germany).

GraphPad Prism software (6.0 Software Version, GraphPad Software, San Diego, CA, USA) was used for statistical analysis. One-way ANOVA method and Bonferroni correction were applied, and statistically significant values were considered for *p* < 0.05.

#### 2.3.3. Encapsulation Efficiency, (EE) of the 5ASA

The EE of 5ASA was determined by the amount of the drug encapsulated in the hydrogel beads using a UV–VIS-NIR spectrophotometer, the UV 3600 (Shimadzu, Kyoto, Japan) with a quartz cell with a light path of 10 mm. The UV spectra were measured at λ = 330 nm. A calibration curve prepared with concentrations of 5ASA between 0.001 and 0.05 mg/mL was used for 5ASA assay.

The EE was calculated using the following equation, Equation (1) [19]:

(1) $$\mathrm{EE}\;(\%)=\frac{\mathrm{Loaded}\;\mathrm{amount}\;\mathrm{of}\;\mathrm{the}\;5\mathrm{ASA}}{\mathrm{Total}\;5\mathrm{ASA}\;\mathrm{amount}}*100$$

#### 2.3.4. Drug Release Study

The drug release profile of the 5ASA from the hydrogel beads was studied in a fully automated dissolution bath USP Apparatus 1 (708-DS Agilent) connected by an auto controlled multi-channel peristaltic pump (810 Agilent) to a UV–VIS spectrophotometer (Cary 60) with 1 mm flow cell (Agilent Technologies, Inc, Santa Clara, CA, USA) and UV dissolution software (Dissolution UV.Ink software, Cary WinUV, Agilent Technologies, Inc, Mulgrave, Victoria, Australia). The drug release studies were conducted in a dialysis membrane bag, where a certain amount of air-dried drug-loaded beads was introduced with 5 mL of buffer solution consisting of simulated gastric fluid (SGF) and pH 1.2 and was first immersed in this medium followed, by their introduction into the simulated intestinal fluid (SIF), pH = 6.8. The dialysis membranes with the hydrogel beads were submersed in 200 mL buffer solution at 37 °C, and the spindle rotation speed was 75 rpm.

#### 2.3.5. Particles Size Analysis

The size and size distribution of particles were measured by a Mastersizer 3000, (Malvern Panalytical Ltd, Worcestershire, Malvern, UK) using the medium volume automated dispersion unit. Samples were dispersed in ultra-pure water (refractive index of 1.330), and the DLS investigations were done at room temperature. For each sample, five measurements were then performed with 2 s delay between them, and the results are shown as mean ± SD.

#### 2.3.6. Morpho-Structural Characterization of Hydrogel Beads

The morpho-structural characterization of hydrogel beads was performed using scanning electron microscopy (SEM) and micro-CT (µCT).

For the micro-computer tomography analysis, Bruker µCT 1272 high-resolution equipment was employed. One particle from each composition was scanned without a filter, the source voltage was set at 80 kV, and the current intensity was set at 125 µA, while the exposure per frame was set at 1200 ms. The scanning procedure was conducted while each sample was rotated by 180°, with a rotation step of 0.15°. A total of 4 frame acquisitions were used to average each individual slice. For the 8 types of particles, the image pixel size was fixed at the value of 500 nm (metric size to pixel size equivalency), while the resolution of a projection was 4904 × 3280 pixels. Tomograms were reconstructed from the raw data in Bruker NRecon 1.7.1.6 software (Bruker, Kontich, Belgium).

Generally, beam hardening correction was set to 40, ring artefact reduction was set to 9, and smoothing was set to 1. Reconstructed datasets were rendered in CTVox (Bruker), while the numerical analysis of the objects was performed using CTAn 1.17.7.2 software (Bruker, Kontich, Belgium).

The surface morphology of the loaded and unloaded drug particles was evaluated using Quanta Inspect F50 Scanning Electron Microscopy (SEM) (FEI, Hillsboro, OR, USA). Prior to testing, the air-dried beads were coated under a vacuum with a thin gold film in order to protect the particles.

## 3. Results and Discussion

### 3.1. Fourier Transform Infrared Spectrometry (FT-IR)

FT-IR spectrometry analysis was performed on drug-loaded CS-TPP beads as well as on neat samples and 5ASA loaded CS-TPP-kCG hydrogel beads in order to evaluate the presence of the drug and to investigate the interactions that occur between the different components of the particles.

**Figure 2:**
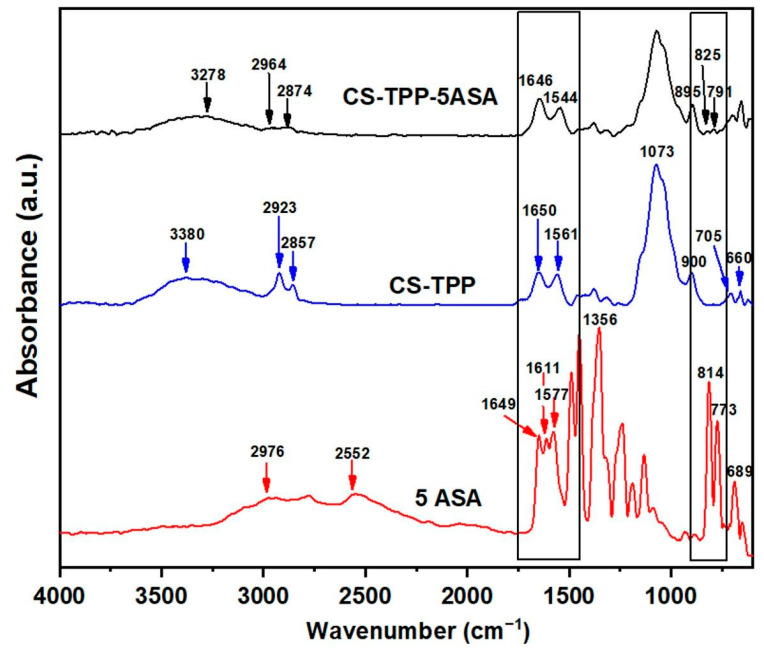
FT-IR spectra of neat and drug loaded CS-TPP hydrogel beads and 5ASA.

The FT-IR spectra of plain CS-TPP particles and drug loaded beads are shown in Figure 2, alongside with the spectrum of 5ASA. The 5ASA spectrum has been recorded and exhibits the absorption bands of the aromatic ring at 2976 cm^−1^ for C–H stretch [33,34]. The FT-IR spectra shows characteristic peaks at 2552 cm^−1^ for intramolecular hydrogen bonding with a carboxyl group and at 1649 cm^−1^ for the C=O stretch, while the band at 1611 cm^−1^ is attributed to the bend vibration of NH and the peak at 1577 cm^−1^ corresponds to the C=C– vibration [19,34,35]. The peak at 1356 cm^−1^ is for the C–N stretch [35], whereas the peaks from 689–814 cm^−1^ are assigned for C–H bond out of plane bending [18,33,35].

The FT-IR spectrum of CS-TPP beads illustrates the glycosidic bonds at 1073 cm^−1^, while the peak at 3380 cm^−1^ is assigned to the vibration of the O–H stretch [27,35,36]. The asymmetrical vibration of methylene and the symmetrical stretch of C–H from CS are assigned at 2923 and 2857 cm^−1^ [17,34]. The characteristic absorption band for the P–O–P asymmetric stretching is at 900 cm^−1^. The bands at 1650 cm^−1^ and 1561 cm^−1^ are attributed to the carboxyl stretching vibration from amide I and N-H bending and the C–N stretching vibrations from amide II [37]

The FT-IR spectrum of CS-TPP hydrogel beads loaded with 5ASA presents the corresponding peaks from the CS-TPP particles.

The drug presence (5ASA) in the hydrogel beads was proved by the increase of the peak intensity at 1646 cm^−1^, which can be attributed to the carboxyl stretching vibration from 5ASA structure.

The appearance of new peaks at 825 and 791 cm^−1^ (attributed to C–H out of plane bending vibrations) in Figure 2 represents additional proof of drug loading.

**Figure 3:**
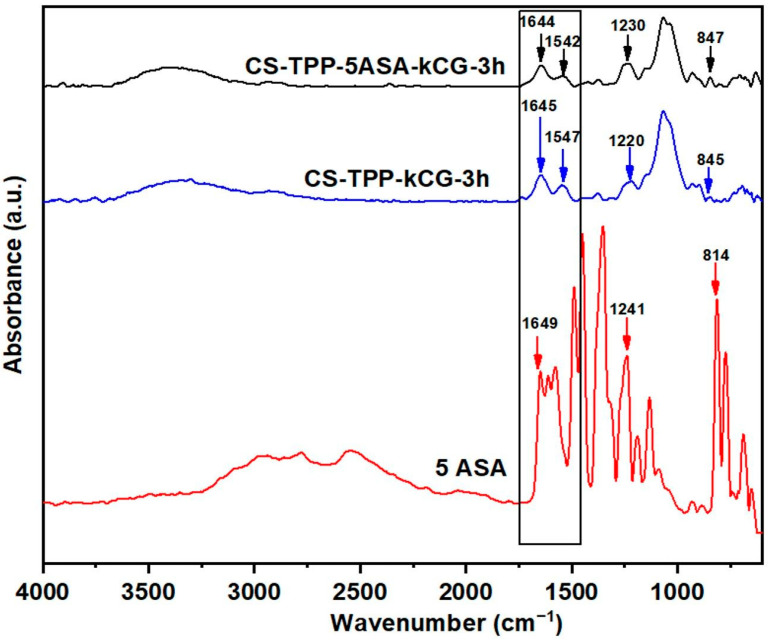
FT-IR spectra of drug loaded and unloaded CS-TPP-kCG-3h particles and 5ASA.

The FTIR results indicate the presence of a kCG layer on the CS-TPP particle surface with the shifting of the peak from 1561 cm^−1^ (Figure 2) to 1547 cm^−1^, proving the formation of a CS-kCG polyelectrolitic complex through the interaction of the amino groups from CS with kCG (Figure 3).

**Figure 4:**
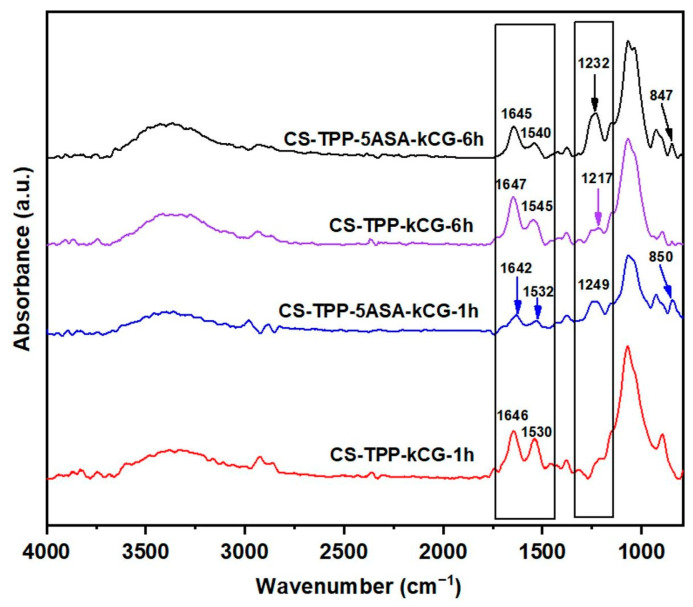
FT-IR spectra of blank, loaded CS-TPP-kCG-1h and CS-TPP-kCG-6h.

This peak shift attributed to amide II vibration was detected in all of the CS-TPP-kCG beads FT-IR spectra (Figure 4). The loading drug in the CS-kCG bicomponent particles was confirmed by the increase of the peak intensity assigned to the carboxyl group stretching vibration in all of drug loaded CS-TPP-kCG beads (1642–1645 cm^−1^) and by the presence of the C_aromatic_-N stretching vibration (1230–1249 cm^−1^) from the drug structure.

Additionally, the new peak at ~850 cm^−1^ attributed to C–H out of plane bending vibrations confirmed the encapsulation of 5ASA.

### 3.2. Encapsulation Efficiency (EE) of 5-Aminosalicylic Acid

The encapsulation efficiency (EE) of the 5ASA in the CS-TPP hydrogel beads prepared with different CS:TPP weight ratios at several crosslinking times are presented in Table 1. The EE was tested for the formulations CS:TPP (1/5 and 1/10).

The EE was obtained in the range of 15% to 40%. The highest drug encapsulation of 40% was given for the beads formulated with CS-TPP = 1:5 and the crosslinking period of 15 min, which means that the drug content in this type of particle is higher. The lowest EE was found for the CS-TPP = 1:10 with the same crosslinking time of 15 min.

After the equilibrium was reached between the ions in the TPP solution and those of the CS needed to obtain the particle*,* the drug diffused from the CS-TPP particles due to its electrostatic interactions with the crosslinking solution determined by the zwitterion nature of 5ASA.

According to Q-X Wu et al. [15], 5ASA might be trapped in a crystal form in a polyelectrolyte complex consisting of CS and TPP due to the pH in an aqueous solution (pH = 4.0, pH = 6.0), in consideration with the solubility of the drug, which is enhanced at pH < 2 and pH > 5.5. The drug content in the CS-TPP-5ASA-kCG hydrogel beads was studied using the UV–VIS spectra of the KCl solution but, it did not contain drug loss from the particles due to the k-CG layer. The explanation for this might be that the negatively charged SO_4_^2−^ groups from kCG presented electrostatic interactions with the NH_3_^+^ group of the CS maintaining the drug trap, so the EE is considered to be the one from the polyelectrolyte complex CS-TPP preparation [29].

### 3.3. Drug Release Study

**Figure 5:**
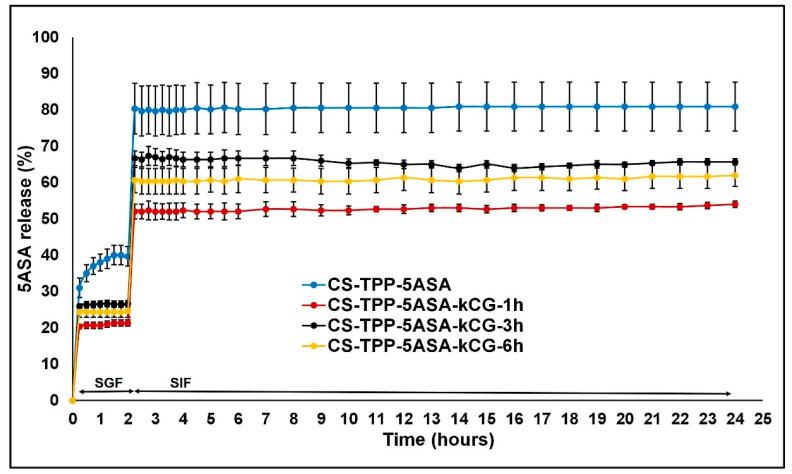
5ASA release profiles from CS-TPP and CS-TPP-kCG under simulated conditions of drug passage through the gastrointestinal tract at 37 °C.

The release profile of the 5ASA from the hydrogel beads was performed by placing a certain amount of air-dried beads in different dissolution media. At first, the samples were suspended for 2 h in SGF (pH = 1.2) and then for 22 h in SIF (pH = 6.8). The amount of 5ASA released from the hydrogel beads was assessed by automatically taking an aliquot of the solution at the determined time intervals and measured using UV spectroscopy (λ = 305 nm at pH = 1.2 and λ = 330 nm at pH = 6.8). The test was performed in triplicate, and the results are displayed in Figure 5.

**Figure 6:**
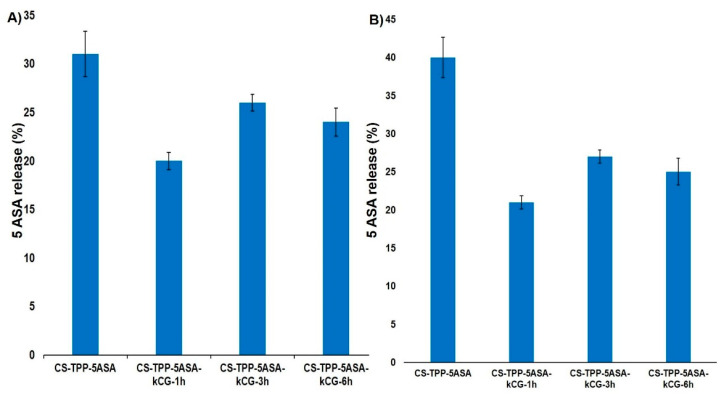
(**A**) Burst release at 15 min and (**B**) drug release after 2 h from the hydrogel beads in SGF at 37 °C.

Both chitosan and k-carrageenan are hydrophilic polymers and allow the advancement of the solution to enter the microspheres and enhance the solubility of the drug. The release profile of the 5ASA from the hydrogel beads is shown in Figure 5 and is defined by several processes. First, it depends on the interactions between the polymers, the solubility of the drug, and the beads in the SGF and SIF solutions as well as swelling behavior [38]. In the first 15 min, in the simulated gastric conditions, a burst release of the drug was observed due to the protonation and high solubility of CS, especially for the CS-TPP particles, increasing the swelling rate [17,27,39], but the presence of the kCG layer significantly reduced this effect for all of the CS-TPP-kCG particles, as illustrated in Figure 6A). The highest reduction of this burst, by almost 10%, is in the hydrogel beads that were kept in the kCG solution for 1 h. By the end of the 2 h period spent in this medium, the drug release from the CS-TPP particles increased, while the drug release from the rest of the particles only changed by 1% (Figure 6B).

This release profile may be attributed to the fact that kCG will remain negatively charged (SO_4_^2−^) and will interact with the oppositely charged groups of CS and will limit the diffusion [40]. Coating the CS-TPP-5ASA particles with the kCG layer protected the drug release from the acidic medium, and the solubility of the kCG is reduced, and the swelling rate decreases [27], protecting the CS-TPP core. Due to the zwitterion nature of 5ASA, we can consider that at pH = 1.2, two interactions can coexist between the drug and the polymers. The electrostatic repulsions between the protonated amino groups of CS and of the protonated drug and an electrostatic interaction between the groups with opposite charges from kCG (SO_4_^2−^) and 5ASA occur, which reduces the fast release of the drug.

**Figure 7:**
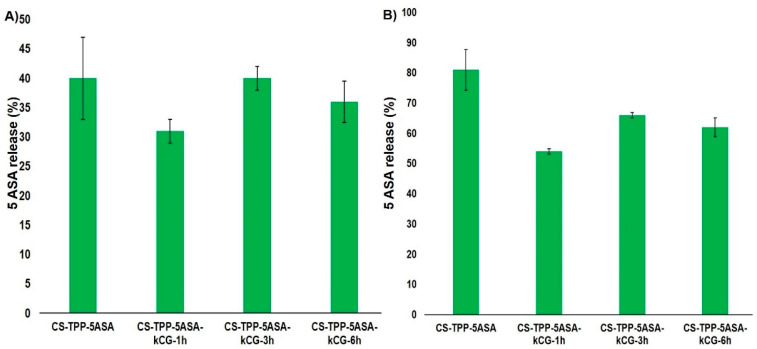
(**A**) Burst release at 15 min after immersion in SIF and (**B**) drug release from the hydrogel beads after 22 h in SIF at 37 °C.

When the medium is changed from acidic to alkaline with a phosphate buffer saline pH = 6.8, a burst release of the drug is also observed to be caused by the sudden transition of the beads into an environment with a different pH, damaging the drug delivery system [41]. However, the influence of the kCG layer helped to decrease this release and the lowest value, 31%, is found to be for the particles that were kept for 1 h in kCG solution (Figure 7A), compared to value of 40% in the hydrogel beads without kCG. After 24 h, the release of the 5ASA from the CS-TPP particles continued to increase up to 81% due to the effect of the acidic medium in which it stayed for 2 h, and there were no interactions between the deprotonated drug and CS. However, in alkaline solution, the sulfate groups of kCG remain negative, reducing the solubility of the polymer and increasing the swelling rate, which causes the electrostatic repulsion between the polymers to appear, and because of the canceling of the CS amino group [27,40], the release of the drug from the CS-TPP-5ASA beads coated with kCG is lower.

Due to these electrostatic forces, we would expect an enhancement in the drug release, but according to the studies of Mladenovska [39], the 5ASA solubility at this pH is low. The release profile of the 5ASA from the Alg/CS-5ASA system was similar because of the electrostatic forces established between the alginate and the drug charged groups [39]. Brions et al. [40] reported that in a CS/kCG complex, kCG endures a helix–coil transition, which makes this system more stiff, causing the slow release rate of the drug. Again, the particles with 1 h of kCG coating had the lowest drug release (Figure 7B).

### 3.4. Particles Size Analysis

Dynamic light scattering (DLS) was employed to measure the size and the distribution of the resulting monodisperse particles in aqueous medium to obtain the hydrodynamic size (Table 2).

**Figure 8:**
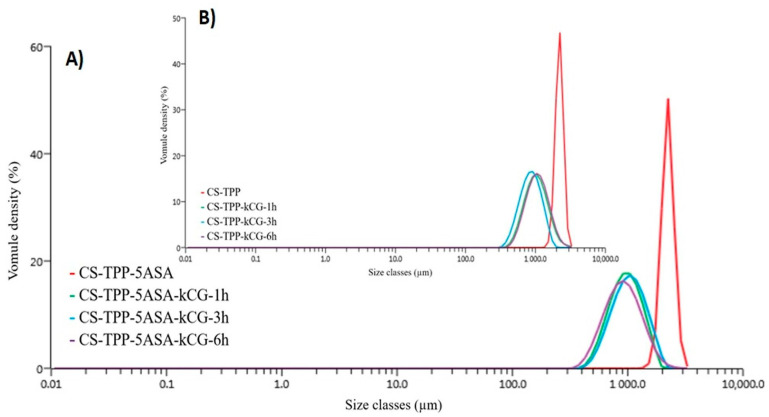
Volume-based particle distribution of CS-TPP-kCG loaded with 5ASA and blank.

Particle size distribution is based on statistical parameters presented as D-values (Dv10, Dv50, Dv90) and SPAN. D-values represent the particle diameter where the sample volume exists at 10, 50, or 90% below a certain size. The other statistical parameter, SPAN, is calculated by (Dv90-Dv10)/Dv50 [42,43,44]. From the results presented in Table 2, it can be observed that by coating the CS-TPP microspheres with a layer of kCG, the particle size decreased because of the development of the polyelectrolyte complex between the two biopolymers, which was also proven by the FT-IR analysis. This decay in size is not correlated with time spent in the kCG solution. Moreover, by adding the drug, no significant difference was found in particle size and distribution compared to the blank ones, which indicates that loading the drug in the hydrogel microspheres did not considerably influence their size. The values are more notable in the case of the CS-TPP-kCG core-shell formulation for the times of 3 and 6 h in the kCG solution. The particle size increased when the carrageenan layer was added in A.V. Briones’s study [40], but with two ways of essential oil encapsulation; in the first method, CS was crosslinked with glutaraldehyde and layered with kCG microspheres, and in the second method, in the CS/kCG polyelectrolyte complex microbeads created by C. Dima et al. [45], the size of the dried microparticles decreases with the growth of the kCG content due to the kCG property that allows it to form a more compact network then CS. Figure 8 shows a monodisperse particle size distribution for the designed particles.

### 3.5. Morphology Characterization of Hydrogel Beads

µCT was employed to assess the morphology of the particles in order to establish the influence of the kCG layers, the crosslinker agent, and the encapsulation of drug. One of the advantages of µCT is its ability to obtain images rapidly and in a non-destructive manner from the surface and from the interior of scanned objects in three dimensions [46].

**Figure 9:**
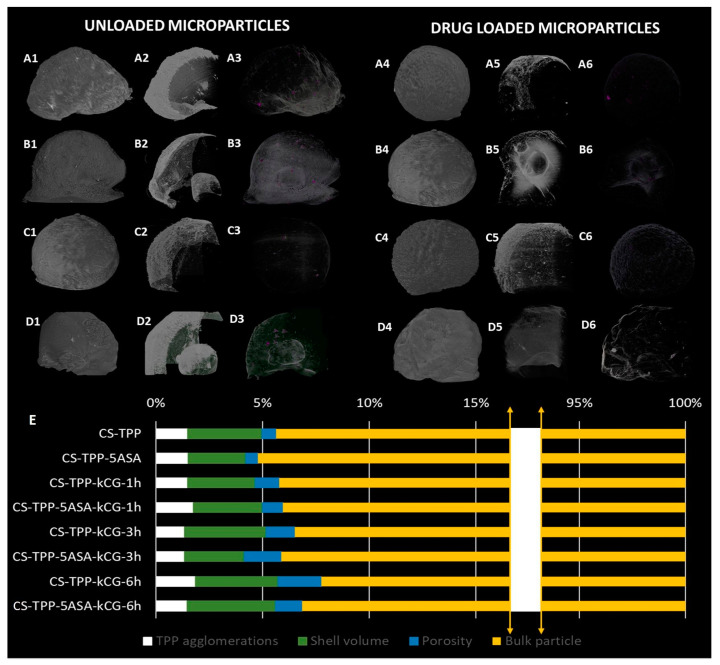
Morphological characterization by means of µCT of the unloaded (**A1–A3**,**D1–D3**) and drug-loaded (**A4**–**A6**,**D4**–**D6**) microparticles, while in the (**E**) section of the image the volume ratios of TPP particles in white, shell volume in green, porosity in blue and the rest of the particulate specimen in yellow are plotted. In the dataset, full volume of the drug free particle is depicted for (**A1**) CS-TPP, (**B1**) CS-TPP-kCG-1h, (**C1**) CS-TPP-kCG-3h, (**D1**) CS-TPP-kCG-6h. In the section, cross-sectional views for the visualization of the particles polymeric shell is illustrated for (**A2**) CS-TPP, (**B2**) CS-TPP-kCG-1h, (**C2**) CS-TPP-kCG-3h, (**D2**) CS-TPP-kCG-6h. In the subdivision, TPP agglomeration within the microparticle volume is preferentially illustrated for (**A3**) CS-TPP, (**B3**) CS-TPP-kCG-1h, (**C3**) CS-TPP-kCG-3h, (**D3**) CS-TPP-kCG-6h. Meanwhile, in the dataset, full volume of the drug-loaded particle is depicted for (**A4**) CS-TPP-5ASA, (**B4**) CS-TPP-5ASA-kCG-1h, (**C4**) CS-TPP-5ASA-kCG-3h, (**D4**) CS-TPP-5ASA-kCG-6h. In the (**A5**–**D5**) section, cross-sectional views for the visualization of the particles polymeric shell is illustrated for (**A5**) CS-TPP-5ASA, (**B5**) CS-TPP-5ASA-kCG-1h, (**C5**) CS-TPP-5ASA-kCG-3h, (**D5**) CS-TPP-5ASA-kCG-6h. In the (**A6**–**D6**) subdivision, TPP agglomeration within the microparticle volume is preferentially illustrated for (**A6**) CS-TPP-5ASA, (**B6**) CS-TPP-5ASA-kCG-1h, (**C6**) CS-TPP-5ASA-kCG-3h, (**D6**) CS-TPP-5ASA-kCG-6h.

The particles were randomly chosen from each group and analyzed in their entirety. For the 3D analysis in CTAn, the tomograms were subjected to an image processing task list consisting of thresholding, to singularly separate the polymer phase walls from its inorganic fillers/pores/polymer shell; despeckling, for the removal of remnant scanning artefacts; and 3D analysis for the numeral quantification of the TPP particles/agglomeration volume. No other image processing technique was applied. Numerical results rendered in Figure 9 depict the inorganic phase percentage of each object.

Figure 9 displays the global appearance and some specificities of the microparticles from the blank and the drug loaded batches. To begin with, the particle morphology was not influenced by the addition of the drug in either specimen (blank or kCG coated sets) due to the low amount of the drug. The scanned particles exhibit irregular morphologies, cvasi-ellipsoidal shapes, with a flattened area generated as a result of the drying process on the plane surface. Overall, the aspect of the particle is rough, with uneven topographical particularities. A tendency towards the patterning of a more wrinkled surfaces emerges in the case of the samples immersed in kCG solution for more than 3 h, with a maximum achieved after 6 h (Figure 9D). The cross-sectional images in Figure 9 illustrate the presence of the polymeric shell based on the contrast within each tomogram. A thresholding grey tone was identified for the samples batch and considering it, the preferential rendering of the outer region of each particle was achieved. By rendering the core of the particle transparent, the shell obtained after the immersion procedure could be distinctly illustrated. Compared to the control particles, where the contrast with the air is still strong, the core–shell particles exhibit an interesting dense crust on their outer region that has a more irregular surface compared to the uncoated beads. In a similar fashion, the TPP agglomerations were pinpointed, and to better highlight them, a coloring procedure was applied (the inorganic phase is preferentially depicted in shades of violet).

CTAn software enabled the quantitative analysis of the samples and a better understanding of some of the particle features. Based on the achieved contrast, the separation between the core of the particles and their shell/pores/TPP agglomerations was achieved. Building on the pixel size value, the algorithms used were able to provide us with quantitative values for the volumes of the pores, shell, and inorganic phase agglomerations. These percentage values are plotted in Figure 9E. Briefly, the volume of the inorganic phase amounts to maximum values of 1.7%, the pores total at most 2%, while the shell represents up to 4.1% of the total volume of each scanned specimen.

**Figure 10:**
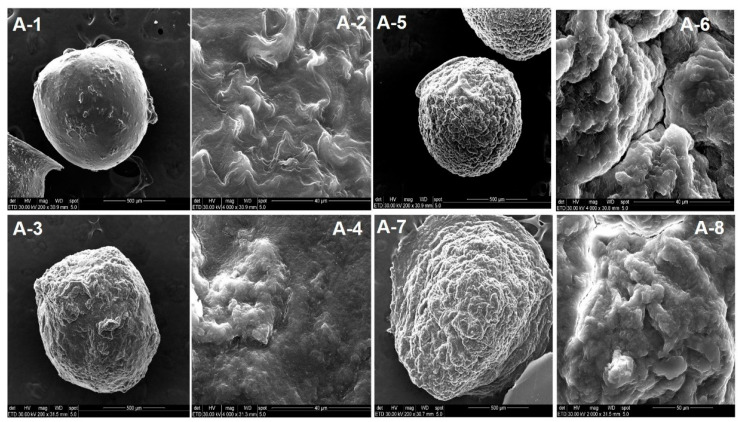
SEM images of the unloaded (**A-1**–**A-8**) hydrogel beads. Figures **A-1** and **A-2** display CS-TPP particles and their surface details. Layered CS-TPP beads with kCG for 1 h, 3 h, and 6 h are illustrated in figures **A-3**, **A-5** and **A-7**, respectively, while their detailed surface textures are depicted in figures **A-4**, **A-6** and **A-8**.

**Figure 11:**
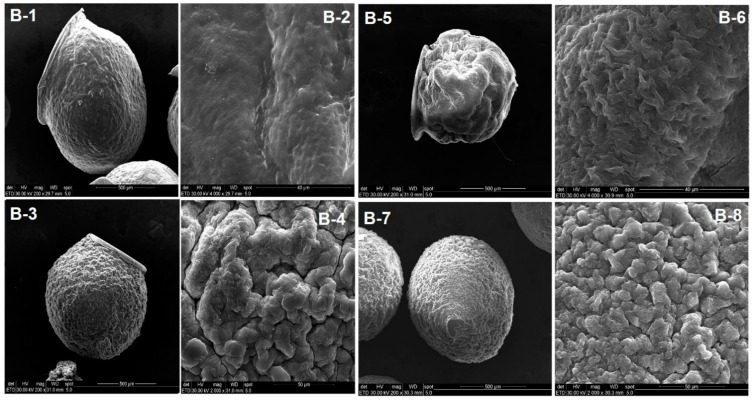
SEM images of the loaded (**B-1**–**B-8**) hydrogel beads. Figures **B-1** and **B-2** display loaded CS-TPP particles and their surface details. Layered loaded CS-TPP beads with kCG for 1 h, 3 h, and 6 h are illustrated in figures **B-3**, **B-5**, and **B-7**, respectively, and their detailed surface textures are depicted in **B-4**, **B-6** and **B-8**.

SEM images allow the observation of the morphology of the dried particles and the effect of the kCG outer layer on the CS-TPP core. The images are presented in Figure 10 and Figure 11. As illustrated in Figure 10A-1 and detailed in Figure 10A-2, the CS-TPP particles presented a smooth surface texture compared to the kCG layered hydrogel beads, which had a rough surface regardless of the time spent in the kCG solution (from Figure 10A-3–A-8).

The same morphology was observed for drug loaded particles (Figure 11B-1–B-8). The particle shape was not completely spherical, and it showed a flattened area due to the air-drying process.

### 3.6. Biocompatibility of the Hydrogel Beads

**Figure 12:**
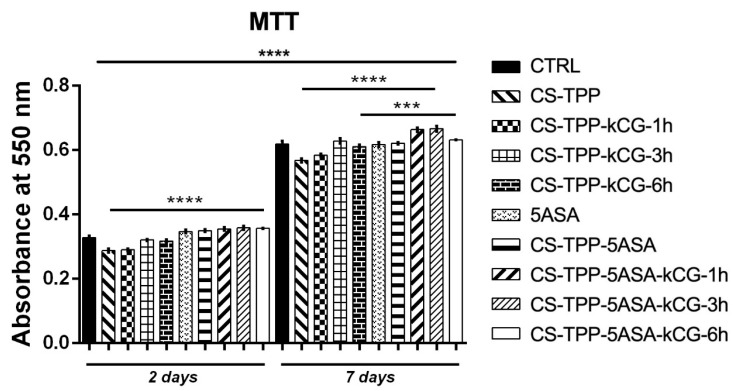
Cell viability and proliferation profile registered after MTT assay for CS-TPP beads at 2 and 7 days of culture. Statistical significance: *** *p* < 0.001, **** *p* < 0.0001.

After 2 days of culture in standard conditions, MTT assay results indicated the overall good viability of the CCD 841 CoN cells cultured in contact with all of the hydrogel beads. The number of viable cells cultured while in contact with the hydrogel beads was similar to the one in the control (CTRL) group. On the other hand, cells that were kept in culture with CS-TPP and CS-TPP-kCG-1h showed a statistically significant (*p* < 0.0001) lower number of viable cells than the other beads. Moreover, cells cultivated in the presence of CS-TPP-5ASA-kCG-1h, CS-TPP-5ASA-kCG-3h, and CS-TPP-5ASA-kCG-6h indicated a slightly increased viability compared to CTRL, although no statistical significance was observed. After 7 days of culture with the beads, high values of viability were observed for the cells kept in contact with all of the tested hydrogels. Cells cultivated in contact with CS-TPP-5ASA, CS-TPP-5ASA-kCG-1h, and CS-TPP-5ASA-kCG-3h particles indicated a statistically significant viability (*p* < 0.0001) in comparison to the CS-TPP, CS-TPP-kCG-1h, and CS-TPP-kCG-3h particles. Similar higher viability results (*p* < 0.001) were noticed for the CS-TPP-5ASA-6h beads when compared to CS-TPP. When comparing the efficiency of 5-ASA to encourage cell proliferation, a statistically significant (*p* < 0.0001) increased proliferation was identified from 2 to 7 days of culture for the cells kept in contact with all of the tested systems. Results are illustrated in Figure 12.

**Figure 13:**
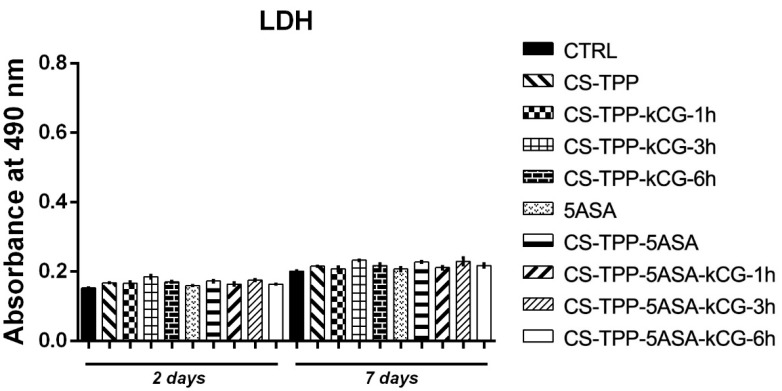
Cytotoxic effect level registered after LDH assay for CS-TPP beads after 2 and 7 days of culture.

Similar levels of material cytotoxicity were indicated by the LDH assay after 2 days of culture in standard conditions. A low number of dead cells was found after they were kept in contact with all of the CS-TPP samples, comparable to the control system. After 7 days of culture, the cytotoxicity of the particles slightly increased, although no statistical significance was identified between the tested particles and the CTRL. Results suggest that CS-TPP, as well as the addition of 5ASA to the material, do not exert a significant cytotoxic effect on the cellular component (Figure 13).

**Figure 14:**
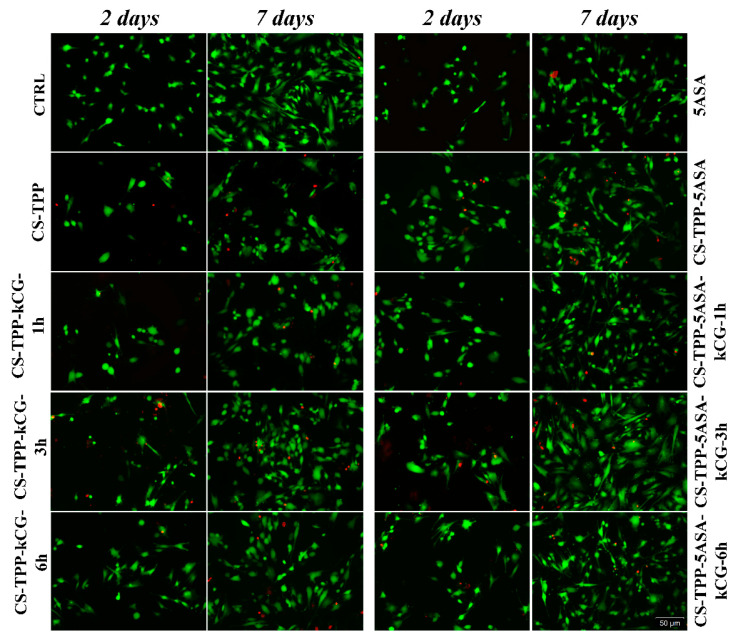
Confocal microscopy of live (green) and dead (red) cells cultured in contact with all of studied particles for 2 and 7 days. Scale bar 50 um.

Live/Dead staining fluorescence microscopy images support the results obtained after the MTT and LDH assays shown in Figure 14. A positive proportion was identified between the live and dead cells. After 2 days of culture, the higher viable cell fraction was determined for all of the CS-TPP-5ASA samples. After 7 days of culture, cells proliferated in contact with CS-TPP, with the highest viable cell proportion found on the CS-TPP hydrogel beads with 5ASA. Moreover, cells cultivated in contact with all of particles displayed an elongated phenotype, suggesting low material cytotoxicity and no negative impact on cell behavior.

## 4. Conclusions

Novel CS-TPP-kCG coated hydrogel particles were successfully obtained using ionic gelation technique with TPP as a crosslinker agent. CS-TPP particles were obtained at different mass weights, and the influence of the kCG layer was studied. The particles were characterized using FT-IR spectrometry and dynamic light scattering. The FT-IR analysis showed the incorporation of the drug and interactions that occur between the different components of the particles. From the DLS measurements, the hydrogel beads display a monodisperse particle size distribution.

Morpho-structural characterization suggested that the obtained hydrogel beads exhibited a smoother texture of the CS-TPP particles than the core-shell beads layered with kCG, which presented roughness, proving the existence of the protective polymer. The presence of 5ASA did not change the morphology of the loaded particles.

The biocompatibility test on the hydrogel beads showed good viability for the CCD 841 CoN cells cultured in contact with all of the beads included for the particles that were suspended in kCG solution. Moreover, a slight increase in cell proliferation was identified even in loaded and unloaded CS-TPP-kCG-6h particles. In addition, cells cultivated in contact with all of the hydrogel beads showed an elongated phenotype, proving low cytotoxicity and no negative impact on cell behavior.

The highest 5ASA encapsulation was ~40% for the beads formulated with a weight ratio of CS/TPP = 1:5 with 15 min crosslinking period, and the kCG coating did not allow the drug to be released from the particles. In the acidic medium of SGF, the kCG layer prevented 5ASA from being released, decreasing the burst release compared to the CS-TPP beads. The same behavior of the CS-TPP-5ASA beads coated with kCG was also seen in the alkaline medium, where the release of the drug is lower. Better results were exhibited in the microsphere coated for 1 h with kCG solution in both SGF (pH = 1.2) and in SIF (pH = 6.8). These results show that the drug loaded CS-TP-kCG particles are a promising candidate for a colon drug delivery system with controlled release.

## Figures and Tables

**Table 1:** Encapsulation efficiency of 5ASA in CS-TPP beads.

Time (Minute)	CS:TPP Ratio	EE (%)	CS:TPP Ratio	EE (%)
15	1:5	40	1:10	15
30		35		25
60		29		21

**Table 2:** Particle size and span value of the hydrogel particles.

Sample Name	Dv10 (µm)	Dv50 (µm)	Dv90 (µm)	Span
CS-TPP	1870 ± 3.02	2200 ± 20.5	2580 ± 45.5	0.333
CS-TPP-5ASA	1900 ± 27.5	2230 ± 50.3	3570 ± 110	0.317
CS-TPP-kCG-1h	637 ± 55.5	1020 ± 105	1630 ± 257	0.987
CS-TPP-kCG-5ASA-1h	624 ± 2.82	957 ± 5.25	1420 ± 7.87	0.834
CS-TPP-kCG-3h	576 ± 31.6	899 ± 47.5	1360 ± 74.8	0.875
CS-TPP-kCG-5ASA-3h	661 ± 4.55	1030 ± 7.50	1560 ± 14.9	0.933
CS-TPP-kCG-6h	661 ± 2.58	1050 ± 4.32	1690 ± 21.2	0.979
CS-TPP-kCG-5ASA-6h	568 ± 2.86	903 ± 1.02	1430 ± 36.0	0.952
